# Differential Immune Activation in Fetal Macrophage Populations

**DOI:** 10.1038/s41598-019-44181-8

**Published:** 2019-05-22

**Authors:** Omar Lakhdari, Asami Yamamura, Gilberto E. Hernandez, Kathryn K. Anderson, Sean J. Lund, Gertrude O. Oppong-Nonterah, Hal M. Hoffman, Lawrence S. Prince

**Affiliations:** Department of Pediatrics, University of California, San Diego, La Jolla, CA, Rady Children’s Hospital, San Diego, San Diego, CA USA

**Keywords:** Alveolar macrophages, Inflammasome

## Abstract

Distinct macrophage subsets populate the developing embryo and fetus in distinct waves. However little is known about the functional differences between in utero macrophage populations or how they might contribute to fetal and neonatal immunity. Here we tested the innate immune response of mouse macrophages derived from the embryonic yolk sac and from fetal liver. When isolated from liver or lung, CD11b^HI^ fetal liver derived macrophages responded to the TLR4 agonist LPS by expressing and releasing inflammatory cytokines. However F4/80^HI^ macrophages from the yolk sac did not respond to LPS treatment. While differences in TLR4 expression did not appear to explain these data, F4/80^HI^ macrophages had much lower NLRP3 inflammasome expression compared to CD11b^HI^ macrophages. Gene expression profiling also demonstrated LPS-induced expression of inflammatory genes in CD11b^HI^ macrophages, but not in F4/80^HI^ cells. Genes expressed in LPS-treated CD11b^HI^ macrophages were more likely to contain predicted NF-κB binding sites in their promoter regions. Our data show that CD11b^HI^ macrophages derived from fetal liver are the major pro-inflammatory cells in the developing fetus. These findings could have important implications in better understanding the fetal inflammatory response and the unique features of neonatal immunity.

## Introduction

Macrophages are key components of the innate immune system. Ubiquitous throughout the body, macrophages defend against harmful microbes, remove apoptotic cells, and promote tissue repair after injury^1^. Upon sensing microbial products, activated macrophages release inflammatory cytokines and chemokines that recruit additional inflammatory cells and cause both local and systemic inflammation. A robust inflammatory response by macrophages can also lead to significant tissue injury^2,3^. Dysregulation of macrophage activation and inflammation contributes to multiple diseases, including atherosclerosis, diabetes, and cancer^4–6^. When playing a trophic role, macrophages can drive tissue fibrosis and tumor growth and metastases^7–9^. Therefore understanding macrophages and their diverse functions could generate new strategies for combatting human disease.

Tissue macrophages are heterogeneous. For example, the lung contains alveolar macrophages, interstitial macrophages, patrolling monocytes, and airway dendritic cells that originate from macrophage/monocyte precursors. The macrophage populations in various tissues appear to arise from unique progenitors during different stages of development. Macrophages populate the embryo and fetus in three successive waves^10,11^. In mice, *Csf1r*+ erythromyeloid progenitors within the yolk sac endothelium give rise to F4/80^HI^ macrophages throughout the embryo beginning at embryonic day 7 (E7). At E9, Myb+ precursors from the yolk sac establish a second site of hematopoiesis within the embryonic liver. From the liver, a second wave of CD11b^HI^ macrophages appear around E12, differentiate into tissue-specific macrophages, and can persist into adulthood. In the lung, these CD11b^HI^ cells differentiate into alveolar macrophages through GM-CSF and TGFβ signaling^12,13^. A third wave occurs after definitive hematopoiesis moves to the bone marrow, partially replacing embryonic yolk sac derived cells with bone marrow derived macrophages via the circulation^14^. While recent publications have shed new light on the origins of macrophage populations, less is known about the functional differences each population plays in immunity.

The fetal and newborn periods present unique immunological challenges. The maternal-fetal immune systems must maintain a tolerant state to prevent attack of the developing fetus by the maternal immune system and also fetal response against maternal antigens^15^. Persistence of tolerance after birth may allow colonization of the newborn with beneficial microbiota and minimize overzealous responses to ingested food antigens^16,17^. The newborn, however, must also be prepared at birth to fend off myriads of microbial pathogens suddenly encountered in the ex utero environment. This unique period in immune development renders newborns susceptible to infection by opportunistic pathogens^18^. In addition, an overzealous immune response in the newborn period, especially in infants born preterm, leads to serious pathology, chronic developmental abnormalities, and lifelong sequelae.

In infants born before 30 wk gestation, macrophage-mediated lung inflammation leads to bronchopulmonary dysplasia^19–23^. A chronic developmental lung disease, bronchopulmonary dysplasia is the most common serious complication of prematurity. In response to mechanical ventilation, infection, and high oxygen exposure, activated lung macrophages stimulate inflammation that prevents normal postnatal airway branching and alveolar formation^24,25^. Both patient and animal studies have implicated macrophage-derived IL-1β as a key cytokine that drives inflammation, injury, and abnormal structural development^22,26,27^. IL-1β release requires both NF-κB mediated *Il1b* transcriptional activation and processing of the pro-IL-1β peptide by inflammasome associated caspase activity. While multiple inflammasome versions exist, the NLRP3-ASC-CASP1 complex is required for lung inflammation and injury in mouse models^22,27^.

Inflammasome function and IL-1β release in the lung is developmentally regulated. Inflammatory stimuli and macrophage activation only impact lung development during later stages of embryonic development^22,28^. The molecular and cellular mechanisms producing this developmental window of susceptibility however are not clear. Here we use flow sorting techniques to isolate macrophage populations from embryonic mice and show that inflammasome function and IL-1β release is specifically restricted to the population of macrophages derived from the fetal liver. By identifying the cell populations that produce tissue inflammation and injury within the developing embryo, our findings will lead to more targeting therapeutic approaches for combatting neonatal inflammatory diseases.

## Material and Methods

### Approvals

Animal experiments were approved by the Institutional Animal Care and Use Committee at University of California, San Diego. All experimental methods were carried out in accordance with the rules, regulations, and guidelines established by the University of California, San Diego Institutional Animal Care and Use Program.

### Mouse strains

C57BL/6 mice were obtained from Harlan Laboratories. *LysM-Cre-NLRP3*^*L351P*^ mice were developed and described previously^29^. The morning of plug identification was defined as embryonic day 0 (E0). Embryos were harvested at E13, E15 or E18. Dissections were performed in cold PBS.

### Fetal macrophage isolation

Fetal organs (lung, liver, brain) and yolk sacs were collected in cold PBS containing EDTA (2 mM) and fetal bovine serum (FBS-2%). Tissues were homogenized and enzymatically digested with collagenase IV (2 mg/ml) for 15 min. red blood cells were lysed with ACK buffer (Life Technologies) for 5 min. Cells suspensions were passed through a 70-μm cell strainer.

### Flow cytometry and cell sorting

Isolated single cell suspensions were first incubated with FC block and Zombie NIR or Aqua live/dead stain (Biolegend) for 15 min in PBS. Conjugated antibodies in staining buffer (2% FBS + 2%EDTA in PBS) were then added for an additional 30 min incubation. For intracellular staining, cells were fixed using IC fixation and permeabilization buffers according to manufacturer’s recommendation (eBioscience). The following antibodies were used for flow sorting: CD45-FITC, CD64-Percp-Cy5.5, F4/80-PE and CD11b-V450. For flow cytometry analysis and intracellular staining, the following additional antibodies were used: CD68-AF647, pro-IL1β-PE-Cy7, TNF-APC-Cy7, NLRP3-APC and CD45-V500.

Flow cytometry measurements were performed on a BD Canto II (BD Biosciences). Sorting was performed on a FACS Aria cell sorter. The following gating strategy was used: doublets were excluded based on forward scatter-A against forward scatter-W, followed by side scatter-A against side scatter-W. Live cells were selected using Zombie LIVE/DEAD stain. CD45+ and CD64+ cells were then selected and further separated based on F4/80 and CD11b expression. Yolk sac derived macrophages express higher F4/80 and lower CD11b levels while fetal monocytes express lower F4/80 and higher CD11b levels. Sorted cells were cultured in suspension in DMEM with 10% FBS with or without gel purified lipopolysaccharide (LPS) from 055:B5 *E. coli* (Sigma L2637). This low-protein preparation of LPS does not appear to activate other Toll-Like Receptors^30,31^.

### RNA isolation and real-time PCR measurement

Cells were homogenized in TRIzol reagent (Invitrogen) and RNA was isolated using Direct-Zol RNA miniprep kit (Zymo Research). After RNA isolation, cDNA was synthesized using first-strand cDNA synthesis kit (Invitrogen). Real-time PCR was performed in CFX96 thermocycler (Bio-Rad) using unlabeled oligonucleotides (IDT) and SYBR Green (Bio-Rad). Gene expression data was presented using the 2^−ΔCt^ method, using TBP as reference gene. The sequences of qPCR primers used are as followed: *Tbp*: 5′-ACA TCT CAG CAA CCC ACA CA and 5′-CTG CTG TGG CAG GAG TGA TA; *Il1b*: 5′-GAC CTG TTC TTT GAA GTT GAC GGA CC and 5′-CAA TGA GTG ATA CTG CCT GCC TGA AG; Il6: 5′-ACA ACC ACG GCC TTC CCT AC and 5′-ACA ATC AGA ATT GCC ATT GCA C; Nlrp3: 5′-GAC CAT CGG CCG GAC TAA AA and 5′-CTT GCA CAC TGG TGG GTT TG; *Pf4*: 5′-CCG AAG AAA GCG ATG GAG ATC T and 5′-CCA GGC AAA TTT TCC TCC CA; *Igf1*: 5′-GTG AGC CAA AGA CAC ACC CA and 5′-.ACC TCT GAT TTT CCG AGT TGC.

### ELISA

For measurements of IL-1β and IL-6 in culture supernatants, we used the Ready-Set-GO ELISAs kits according to manufacturer’s protocol (eBioscience).

### Monocyte depletion

For Ly6C+ monocyte depletion, pregnant mice received 3 intraperitoneal injections of anti-Ly6G/Ly6C (clone RB6-8C5, Bioxcell) antibody or the isotype control (rat IgG2b anti-KLH, clone LTF-2, Bioxcell), starting at E12. For the first injection, mice received 1 mg of antibodies, then 2 mg for the second and third injections. Embryos were collected at E15.

### Gene expression analysis with Nanostring nCounter technology

The nCounter analysis system (NanoString Technologies) was used to assess gene expression by CD11b and F4/80 macrophages. RNA from sorted cells was amplified then hybridized with the Mouse Myeloid Innate Immunity Panel v2 (734 immunology-related mouse genes + 20 internal reference controls) according to manufacturer’s protocol. Processing was performed in the UCSD Stem Cell Genomics Core at the Sanford Consortium for Regenerative Medicine. Data analysis was performed using nSolver 4.0 according to NanoString guidelines. Raw mRNA counts were normalized to account for hybridization efficiency, background noise, and sample content. Genes with mean counts less than 20 were excluded, and differential expression of remaining genes was calculated and represented on a volcano plot, using a nonparametric t-test and correction for multiple testing using the BH false discovery rate (FDR). Mouse single site analysis of conserved transcription factor (TF) binding sites in the list of differentially expressed genes was performed using oPOSSUM 3.0 (http://opossum.cisreg.ca/oPOSSUM3/), using JASPAR core profile and the oPOSSUM gene database as background.

## Results

### CD11b^HI^ fetal lung macrophages are the primary sources of IL-1β and IL-6

**Figure 1:**
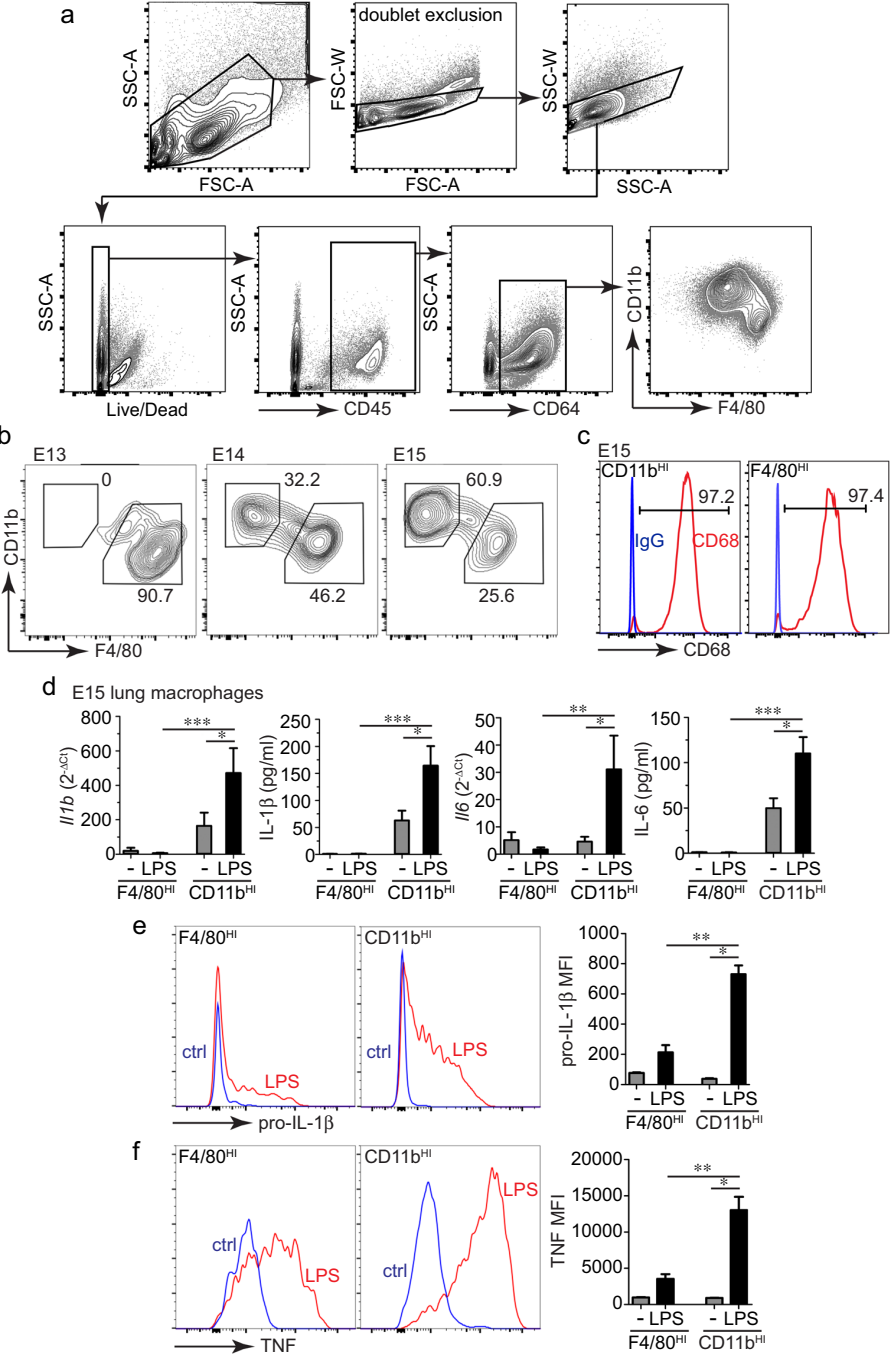
Fetal lung macrophages have differential responses to LPS. (**a**) Gating strategy used to isolate live, CD45+, CD64+ fetal macrophage populations. (**b**) FACS analysis of E13, E14, and E15 mouse lung macrophages (CD45+, CD64+) identified two different macrophage populations beginning at E14 based on relative CD11b and F4/80 expression. Percentage of CD45+, CD64+ cells in each population indicated. (**c**) Both CD11b^HI^ and F4/80^HI^ populations expressed the macrophage marker CD68 (red). Isotype control IgG staining is included as a control (blue), and the percentage of cells expressing CD68 is indicated. (**d**) Using the differential relative expression of CD11b and F4/80, two different macrophage populations were sorted and collected from E15 mouse lungs. After sorting, cells were cultured in suspension under control conditions or in the presence of *E. coli* LPS (O55:B5, gel-purified, 250 ng/ml). After two hours, cells were harvested by centrifugation and solubilized in TRIzol. Media was collected for ELISA. *Il1b* and *Il6* mRNA was measured by real time PCR; IL-1β and IL-6 in the media was measured by ELISA. Compared to F4/80^HI^ macrophages, CD11b^HI^ macrophages expressed higher levels of cytokines both under basal conditions and following LPS treatment (+/− s.e.m.; *P < 0.05, **P < 0.01, ***P < 0.001; n = 5–8). (**e**,**f**) Measurement of IL-1β and TNF expression in fetal lung macrophages by intracellular immunostaining and FACS. Single cell suspensions from E15 mouse lungs were treated with LPS for 2 h followed by fixation, staining, and FACS. LPS induced higher IL-1β and TNF expression in CD11b^HI^ macrophages compared to F4/80^HI^ macrophages (+/− s.e.m.; *P < 0.05, **P < 0.01, n = 3).

Fetal lung inflammation following macrophage activation increases during the later stages of development. Here we tested if this temporal change was due to inflammatory differentiation of resident tissue macrophages or appearance of new pro-inflammatory cells later in development. Using a flow sorting approach (Fig. 1a), we first demonstrated the timing of when different macrophage populations appeared in the developing mouse lung (Fig. 1b). The E13 lung contained primarily F4/80^HI^/CD11b^LO^ (F4/80^HI^) macrophages (with macrophages defined as CD45+/CD64+). These F4/80^HI^ macrophages are derived from myeloid precursor cells in the embryonic yolk sac. Beginning at E14, F4/80^LO^/CD11b^HI^ (CD11b^HI^) macrophages appeared, derived from fetal liver monocytes. The percentage of CD11b^HI^ macrophages increased further at E15. Both CD11b^HI^ and F4/80^HI^ cells expressed the macrophage marker CD68 (Fig. 1c).

To test if yolk sac F4/80^HI^ and fetal liver derived CD11b^HI^ lung macrophage populations had distinct functional properties, we sorted both macrophage populations from E15 lungs and stimulated them with the TLR4 agonist LPS (gel purified from *E. coli*, O55:B5). Importantly, freshly sorted cells were kept in suspension and treated with LPS immediately upon sorting to reduce potential phenotypic changes with cell culture. LPS increased both *Il1b* and *Il6* mRNA expression and IL-1β and IL-6 protein release in CD11b^HI^ macrophages (Fig. 1d). F4/80^HI^ macrophages expressed very low cytokine mRNA levels and did not release detectable amounts of cytokine peptides either under control conditions or upon LPS treatment. We also measured intracellular IL-1β and TNF within LPS-treated E15 lung cell suspensions using FACS (Fig. 1e,f). Similar to our real time PCR and ELISA data, inflammatory cytokine expression was restricted to CD11b^HI^ lung macrophages and not detected in F4/80^HI^ cells. LPS did not significantly increase cell death in either F4/80^HI^ or CD11b^HI^ macrophages as assayed by live-dead staining (not shown). CD11b^HI^ macrophages appearing in the fetal lung after E13 were therefore more pro-inflammatory after LPS stimulation and with higher cytokine expression and release.

### LPS-induced cytokine release is also restricted to CD11b^HI^ macrophages in the fetal liver

**Figure 2:**
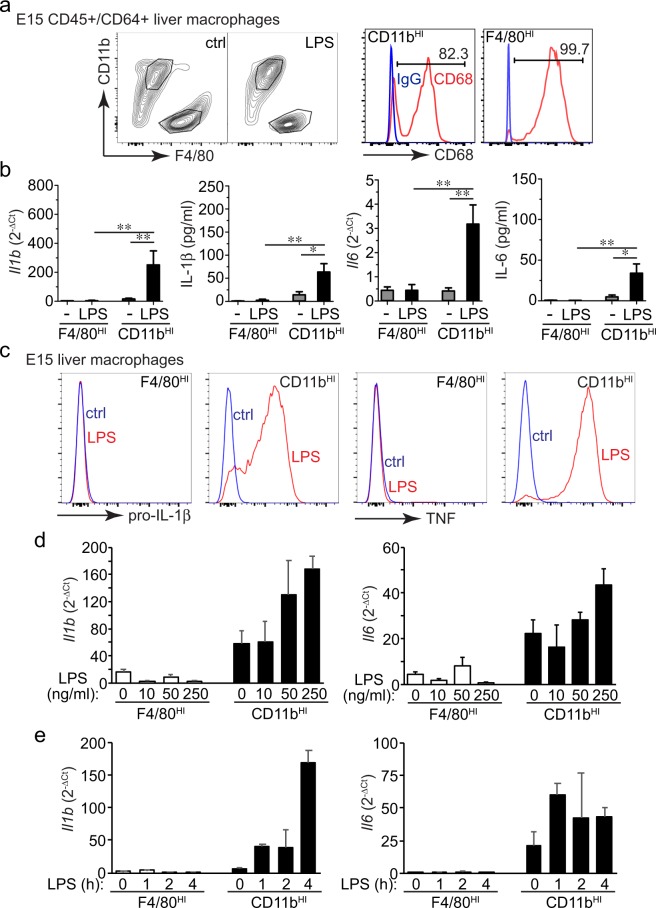
Differential response to LPS between fetal liver macrophage populations. (**a**) E15 fetal mouse livers contain both CD11b^HI^ and F4/80^HI^ macrophage populations that also express CD68 (red). Isotype control IgG staining is included as a control (blue) and the percentage of cells expressing CD68 is indicated. (**b**) In sorted CD11b^HI^ macrophages from E15 liver, LPS increased IL-1β and IL-6 gene expression and cytokine release into the media. However, LPS did not increase expression or release in F4/80^HI^ macrophages. (+/− s.e.m.; *P < 0.05, **P < 0.01, n = 4–7). (**c**) Intracellular immunostaining and FACS analysis only detected pro-IL-1β and TNF in CD11b^HI^ macrophages. F4/80^HI^ cells did not express either cytokine, even following LPS treatment. Data shown are from a single experiment representative of three independent experiments. (**d**) Dose response data measuring *Il1b* and *Il6* expression by real time PCR in CD11b^HI^ and F4/80^HI^ macrophage populations treated for 4 h with 0, 10 ng/ml, 50 ng/ml, or 250 ng/ml LPS (n = 3). (**e**) Time course of the LPS response (250 ng/ml) in both CD11b^HI^ and F4/80^HI^ macrophage populations (n = 3).

To test if the functional differences between macrophage populations were tissue specific, we next isolated F4/80^HI^ and CD11b^HI^ macrophages from E15 fetal liver and measured their LPS response (Fig. 2a). Similar to lung macrophages, LPS stimulated cytokine expression and release in CD11b^HI^ fetal liver macrophages but not in F4/80^HI^ macrophages (Fig. 2b,c). Of note, cytokine expression was lower in E15 liver CD11b^HI^ macrophages compared to CD11b^HI^ cells isolated from E15 lung as measured in Fig. 1. We next compared the dose response and time course of LPS activation between the two fetal macrophage populations. As shown in Fig. 2d, F4/80^HI^ macrophages failed to respond at all LPS concentrations tested. We also did not detect increased *Il1b* or *Il6* mRNA in LPS-treated F4/80^HI^ macrophages over a 4 h time course. These data further confirm the proinflammatory phenotype in fetal CD11b^HI^ macrophages and the resistance to LPS in fetal F4/80^HI^ macrophages.

**Figure 3:**
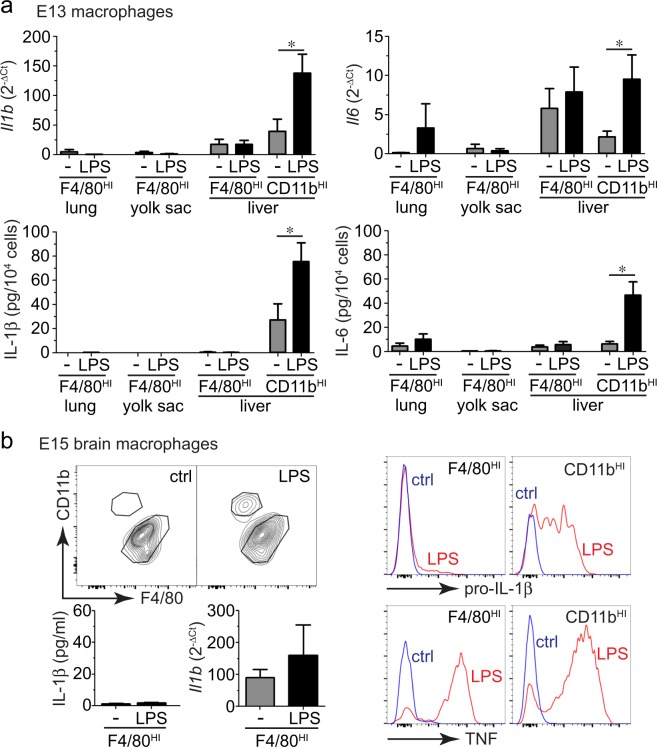
(**a**) LPS responsiveness is only present in CD11b^HI^ macrophages as early as E13. Macrophage populations from E13 mouse lung, yolk sac, and liver were sorted, isolated, and stimulated with LPS. Only F4/80^HI^ macrophages could be isolated from E13 lung and yolk sac. LPS did not increase cytokine expression or release from E13 F4/80^HI^ macrophages (+/− s.e.m.; *P < 0.05, n = 3–7). (**b**) F4/80^HI^ macrophages in E15 mouse brains have a partial response to LPS. The majority of macrophages in E15 brain were F4/80^HI^, and LPS treatment did not increase IL-1β release or mRNA expression (+/− s.e.m.; n = 6–7). Intracellular staining and FACS did demonstrate increased TNF expression in F4/80^HI^ macrophages from E15 brain, but only minimal pro-IL-1β expression (representative data from three independent experiments shown).

To test if the differential response to LPS was present earlier in development, we isolated macrophage populations from yolk sac, liver, and lung at E13 (Fig. 3a). LPS did not induce cytokine expression in F4/80^HI^ E13 macrophages from any of the tissue sources tested. At E13, only the liver contained CD11b^HI^ macrophages, and these cells did express IL-1β and IL-6 following LPS treatment. Interestingly, F4/80^HI^ cells from the E13 liver appeared to express higher basal levels of *Il1b* and *Il6* mRNA compared to macrophages from yolk sac and lung, but cytokine expression did not change with LPS. E13 liver F4/80^HI^ macrophages also did not release cytokine peptides into the media after LPS treatment. While both F4/80^HI^ and CD11b^HI^ macrophages populate most tissues within the developing embryo, the brain contains primarily F4/80^HI^ yolk sac macrophages. LPS treatment of F4/80^HI^ macrophages from E15 brain did not induce IL-1β expression or release (Fig. 3b), similar to the results obtained with other F4/80^HI^ macrophage populations. However F4/80^HI^ brain macrophages did express TNF after LPS treatment. The unique brain microenvironment therefore appears to promote at least a partially competent inflammatory state in F4/80^HI^ macrophages.

**Figure 4:**
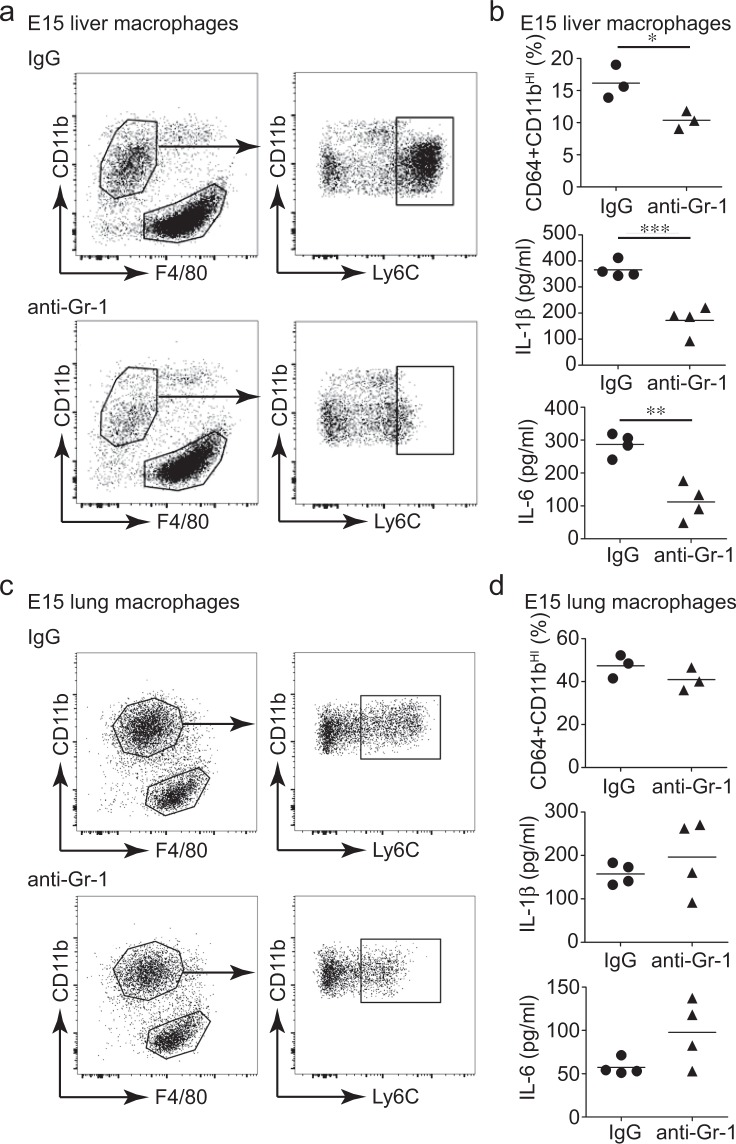
Depletion of CD11b^HI^/Ly6C+ macrophages reduces IL-1β production in E15 fetal liver. Pregnant mice were injected with nonimmune IgG or anti-GR-1 antibody. At E15, macrophages were isolated from fetal liver (**a,b**) and fetal lung (**c,d**) Anti-Gr-1 injection reduced the percentage of CD11b^HI^/Ly6C+ macrophages in the E15 fetal liver (**a**) with a corresponding reduction in IL-1β and IL-6 release following treatment of the total fetal liver cell suspension with LPS (*P < 0.05, n = 3; **P < 0.01, n = 4, ***P < 0.001, n = 4). Anti-Gr-1 injections did not reduce CD11b^HI^/Ly6C+ macrophages from fetal lungs or reduce IL-1β or IL-6 production following LPS treatment.

Based on these data, we hypothesized that CD11b^HI^ macrophages were the primary inflammatory cells in the developing embryo. To test the requirement of CD11b^HI^ macrophages in mediating inflammation, we injected pregnant dams with monoclonal anti-Gr-1 IgG to immunodeplete CD11b^HI^ cells from developing embryos^32,33^. Figure 4 shows that anti-Gr-1 injection reduced the number of Ly6C+/CD11b^HI^ macrophages in the fetal liver at E15. When the total liver cell suspension was treated with LPS, Gr-1 depleted samples had reduced levels of IL-1β and IL-6 release equivalent with the level of depletion (Fig. 4a,b). These data supported CD11b^HI^ macrophages as a major population of cells in the fetal liver releasing IL-1β and IL-6 following LPS treatment. While anti-Gr-1 IgG immunodepleted Ly6C+ cells from the E15 lung, the number of CD11b^HI^ macrophages was unchanged (Fig. 4c,d). LPS stimulated IL-1β and IL-6 release was unaffected by anti-Gr-1 depletion in E15 lung samples, consistent with our hypothesis that CD11b^HI^ macrophages were the main cellular source of IL-1β.

### Tlr4 and Nlrp3 inflammasome expression in CD11b^HI^ fetal macrophages

**Figure 5:**
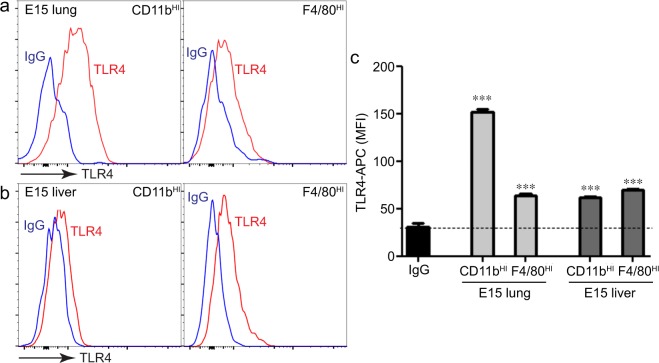
Both fetal macrophage populations express the LPS receptor TLR4. (**a,b**) E15 lung macrophages were immunolabeled with anti-TLR and analyzed by FACS. (**c**) TLR4-APC median fluorescence intensity (MFI) values measured TLR4 surface expression in each macrophage population. While CD11b^HI^ macrophages from E15 lung appeared to express higher levels of TLR4 compared to F4/80^HI^ lung macrophages, the relative expression of TLR4 in liver macrophage populations was similar (+/− s.e.m.; ***P < 0.001 compared to IgG isotype control, n = 4–6).

**Figure 6:**
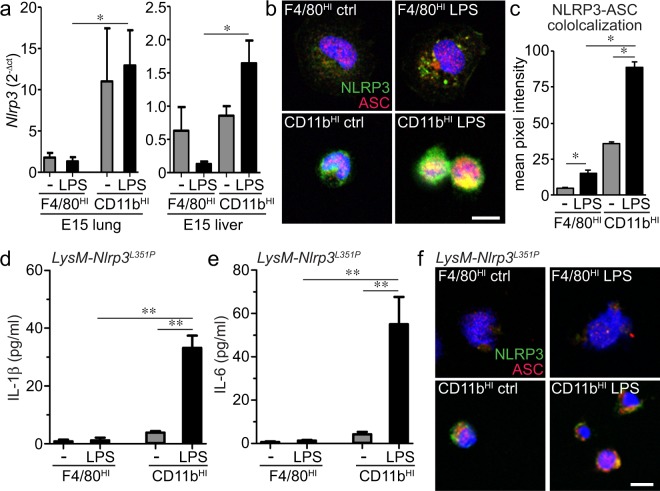
Proinflammatory CD11b^HI^ fetal macrophages express higher levels of the NLRP3 inflammasome. (**a**) Real time PCR measured higher *Nlrp3* mRNA levels in LPS treated CD11b^HI^ macrophages compared to F4/80^HI^ macrophages in both E15 lung and E15 liver (+/− s.e.m.; *P < 0.05, n = 3–5). (**b**) CD11b^HI^ macrophages from fetal liver expressed higher levels of NLRP3 and the linker protein ASC compared to F4/80^HI^ macrophages as visualized by laser scanning confocal microscopy. (**c**) LPS increased NLRP3-ASC colocalization in both macrophage populations. For colocalization measurements, pixel intensities in the red, green, and yellow (colocalized) channels were measured in identically sized regions of interest. Higher pixel intensity was measured in CD11b^HI^ macrophages (+/− s.e.m.; *P < 0.05, n = 3). (**d,e**) Macrophage populations were sorted from E15 *LysM-Nlrp3*^*L351P*^ inflammasome gain of function mice and treated with LPS. IL-1β (**d**) and IL-6 (**e**) were only detected in the media from LPS-treated CD11b^HI^ macrophages (+/− s.e.m.; **P < 0.01, n = 6). (**f**) Expression of NLRP3 and ASC inflammasome components were lower in F4/80^HI^ macrophages from E15 *LysM-Nlrp3*^*L351P*^ mice.

IL-1β expression and release following LPS exposure requires both TLR4 signaling and NLRP3 inflammasome expression, assembly, and activation. We next tested if fetal lung macrophage populations had differential TLR4 or NLRP3 expression. CD11b^HI^ macrophages from the lung did express higher levels of TLR4 compared to F4/80^HI^ macrophages (Fig. 5a,c). However E15 liver macrophage populations expressed similar TLR4 levels (Fig. 5b,c). Potential differences in TLR4 expression therefore did not appear to explain why F4/80^HI^ macrophages failed to express inflammatory cytokines after LPS treatment. NLRP3 expression in the developing lung is restricted to macrophages and increases during the later stages of lung development. CD11b^HI^ macrophages from E15 lung expressed higher *Nlrp3* compared to F4/80^HI^ macrophages (Fig. 6a). LPS increased *Nlrp3* expression in CD11b^HI^ cells with minimal effect on F4/80^HI^ macrophages. Similar results were seen by immunostaining macrophages with antibodies against NLRP3 and the inflammasome linker protein ASC (Fig. 6b). Expression and co-localization were more prevalent in CD11b^HI^ cells. LPS treatment increased co-localization in both macrophage populations, with much higher ASC-NLRP3 co-localized signal in CD11b^HI^ macrophages (Fig. 6c).

We next tested if constitutively active NLRP3 was sufficient to drive cytokine release in both CD11b^HI^ and F4/80^HI^ macrophages. We isolated E15 liver macrophages from *Nlrp3*^*L351P*^ mice expressing a gain of function mutation within the *Nlrp3* gene. At E15, neither macrophage population released significant IL-1β under control conditions (Fig. 6d,e). LPS treatment however stimulated both IL-1β and IL-6 release only in CD11b^HI^ macrophages. F4/80^HI^ macrophages from E15 *Nlrp3*^*L351P*^ mice failed to express high levels of NLRP3 by confocal immunofluorescence, even after LPS treatment (Fig. 6f). *Nlrp3*^*L351P*^ CD11b^HI^ macrophages expressed both NLRP3 and ASC, with expression and colocalization increased after LPS treatment. Consistent with our data in wild type macrophages, these results show that fetal CD11b^HI^ macrophages express NLRP3 inflammasome components and have the capacity to express and release IL-1β upon TLR4 stimulation. F4/80^HI^ macrophages however failed to express NLRP3, preventing their ability to release IL-1β. Coupled with the lack of both *Il1b* and *Il6* mRNA in F4/80^HI^ macrophages, these data suggest that F4/80^HI^ macrophages lack an innate immune transcriptional response as well as the machinery necessary for generating pro-inflammatory activation.

### Differential myeloid gene expression in LPS-treated fetal macrophages

**Figure 7:**
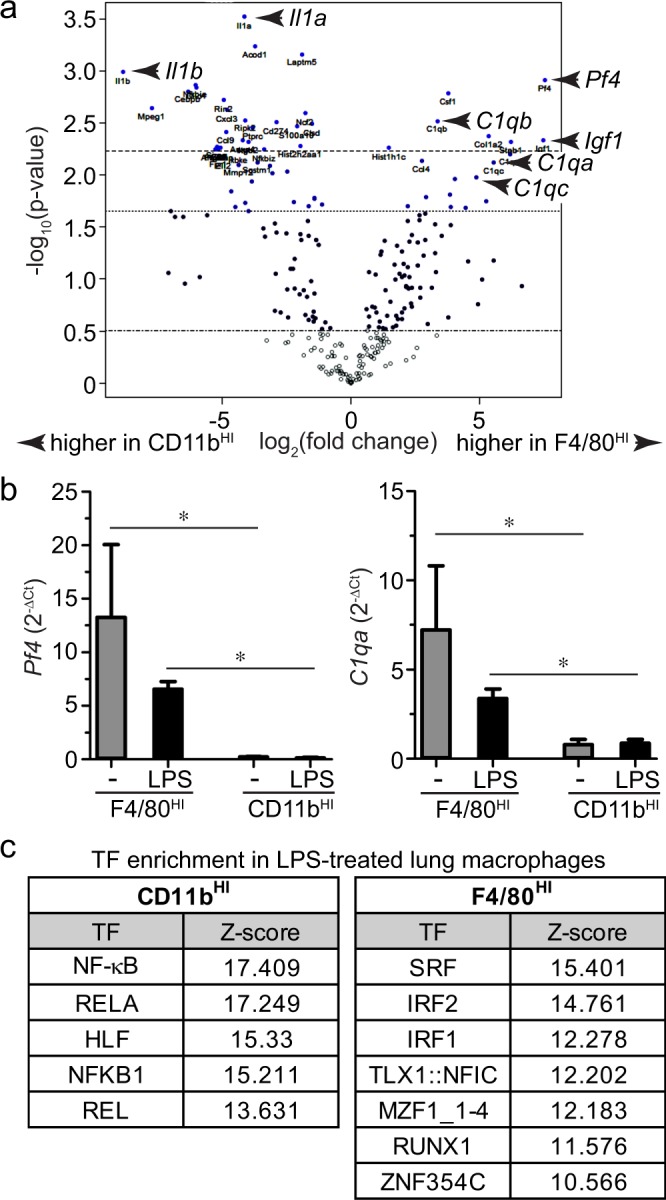
Differential gene expression in LPS-treated CD11b^HI^ macrophages vs. F4/80^HI^ macrophages from E15 mouse lung. (**a**) Volcano plot of myeloid gene expression as measured by Nanostring assay. Genes more highly expressed in CD11b^HI^ samples are shifted to the left. Genes higher in F4/80^HI^ macrophages are shifted to the right. *Il1a* and *Il1b* (higher in CD11b^HI^ macrophages) and *C1qa, C1qb, C1qc, Pf4*, and *Igf1* (higher in F4/80^HI^ macrophages) are noted by arrowheads (n = 3 for F4/80^HI^, n = 4 for CD11b^HI^). (**b**) Real time PCR confirmed higher *Pf4* and *C1qa* expression in F4/80^HI^ macrophages. Neither gene was increased by LPS treatment (+/− s.e.m.; *P < 0.05, n = 3–4). (**c**) Transcription factor binding sites enriched in differentially expressed genes identified using oPOSSUM.

The above experiments focused on *Il1b*, *Il6*, and *Nlrp3* in initially assessing the LPS-induced inflammatory response in F4/80^HI^ and CD11b^HI^ fetal macrophages. To measure expression of additional genes, we used Nanostring nCounter gene expression profiling of 734 myeloid genes in LPS treated F4/80^HI^ and CD11b^HI^ macrophages from E15 mouse lung (Fig. 7a). A total of 89 genes were differentially expressed between the 2 macrophage populations (*log*_2_ fold change >1; adjusted *P* < 0.05). CD11b^HI^ cells expressed higher levels of *Il1a*, *Il1b*, *Ccl9*, and *Cxcl3*, while F4/80^HI^ cells had higher expression of *Pf4* (also known as *Cxcl4*), *Igf1*, and the complement genes *C1qa*, *C1qb* and *C1qc* (Fig. 7a). Real time PCR confirmed higher *C1qa* and *Pf4* expression in F4/80^HI^ macrophages and LPS did not increase expression of either gene (Fig. 7b). To identify the molecular mechanisms of differential gene expression in the two macrophage populations, we examined TF binding sites enriched in LPS-induced genes in CD11b^HI^ macrophages compared to F4/80^HI^ macrophages using Opossum 3.0 (Fig. 7c). Overexpressed genes in CD11b^HI^ macrophages were notably enriched in NF-κB binding sites, while the F4/80^HI^ macrophage gene signature contained motifs predicted to bind SRF, IRF1, and IRF2. In addition, RUNX1 predicted sites in F4/80^HI^ samples were consistent with data showing *Runx1* expression is restricted to yolk sac derived macrophages^34^. In summary, our data suggest fetal macrophage populations have inherent differences in the transcriptional response machinery following exposure to LPS.

## Discussion

Fetal lung inflammation is developmentally regulated, with macrophage activation and cytokine release impacting airway morphogenesis only during the later stages of *in utero* development^22^. Here we show that developmental regulation of lung inflammation is due to the programmed accumulation of pro-inflammatory macrophages in the lung after E15. While we and others have shown macrophages do populate the lung from the initial days of embryonic development^12,23,35^, these early yolk sac derived macrophages appear resistant to pro-inflammatory TLR mediated activation. Our data here clearly demonstrate the functional differences between F4/80^HI^ yolk sac derived macrophages and CD11b^HI^ fetal liver derived macrophages. CD11b^HI^ macrophages express higher levels of inflammasome components and release IL-1β and IL-6 upon LPS treatment, while F4/80^HI^ macrophages fail to release these inflammatory mediators that play important roles in injury and disease.

Macrophage populations may share common functional properties in the moments following their differentiation from myeloid progenitors during fetal hematopoiesis. Upon trafficking to the circulation and into various tissues, the local microenvironment then shapes macrophage biology and promotes specialized, tissue specific functions^11,36^. While the adult lung contains multiple immune cell populations including dendritic cells and lymphocytes, many of these cells appear after birth^37,38^. However we still have not determined the relative heterogeneity within the various fetal macrophage populations studied here. At E15 when fetal liver derived CD11b^HI^ macrophages are first trafficking to various tissues, cells isolated from the fetal lung had a slightly higher expression of inflammatory cytokines compared to cells still residing within the liver. The high levels of GM-CSF present in the lung could mediate these differences^39^. F4/80^HI^ cells from the yolk sac, liver, and lung remained resistant to LPS stimulation. The strikingly different situation identified for F4/80^HI^ cells is the developing fetal brain. Macrophages in the fetal brain are primarily F4/80^HI^ cells from the yolk sac, and these cells eventually differentiate into microglia^34,40^. Not only do brain microglia have inflammatory potential, they also play a key role in forming mature synapses^41–43^. Interestingly, F4/80^HI^ macrophages from the E15 fetal brain did respond to LPS by increasing TNF expression. However they appeared to lack functional NLRP3 inflammasome expression and therefore failed to release IL-1β. Identifying the factors within the brain that give F4/80^HI^ cells their unique functional properties could provide important insight into both macrophage biology and neuroinflammatory disease processes.

The functional differences between fetal macrophage populations suggest unique molecular regulatory mechanisms. While both macrophage populations expressed similar levels of the LPS receptor TLR4, F4/80^HI^ macrophages did not express the same levels of NF-κB dependent genes after LPS treatment. Therefore F4/80^HI^ macrophages could have reduced expression of proteins required for the TLR-IKK-NF-κB or MAPK signaling pathways. Differential transcription factor expression or epigenetic chromatin modifications between CD11b^HI^ and F4/80^HI^ macrophages could also explain why LPS generated unique responses in each cell population. Our informatics analysis clearly identified NF-κB promoter sites activated in CD11b^HI^ macrophages that were apparently not accessible in F4/80^HI^ cells. Ongoing experiments are exploring the potential upstream mechanisms responsible for these differences.

If CD11b^HI^ cells mount inflammatory responses to microbes in the developing embryo, then what are the roles of F4/80^HI^ macrophages? F4/80^HI^ cells migrate throughout the entire embryo very early in development. The ubiquitous locations and lack of TLR-mediated inflammatory function suggest they play a role in tissue morphogenesis or homeostasis. Macrophages remove dying and apoptotic cells from both developing and mature tissues^44^. Multiple receptors on phagocytic cells bind outer leaflet phosphatidylserine on apoptotic and necrotic cells^45^. The complement component C1q promotes phagocytosis of dying cells by macrophages^46–48^. We measured higher levels of the C1q genes *C1qa, C1qb*, and *C1qc* in F4/80^HI^ macrophages from fetal lung compared to CD11b^HI^ macrophages. These data suggest that yolk sac derived F4/80^HI^ macrophages play important roles in removing apoptotic or necrotic cells during tissue morphogenesis. F4/80^HI^ macrophages also expressed IGF-1, a major promoter of both developmental morphogenesis and tissue repair and regeneration. Macrophage IGF-1 contributes to muscle repair following injury^49^. In the lung, macrophage phagocytosis of apoptotic cells in the lung releases IGF-1, which then stimulates additional phagocytic function in adjacent airway epithelial cells^50^. The transcription factors MafB and C/EBPγ were both detected in Nanostring analysis of F4/80^HI^ macrophages, and both factors drive anti-inflammatory cell phenotypes^51,52^. Data therefore suggest that anti-inflammatory fetal F4/80^HI^ macrophages could be tasked with removing damaged cells and cellular debris during morphogenesis while pro-inflammatory CD11b^HI^ macrophages respond to microbial products and innate immune activators.

The unique functions of fetal macrophage populations could impact disease processes. Early in development, the lack of proinflammatory macrophages prevents a robust innate immune response. Upon appearance of activation competent CD11b^HI^ macrophages in various tissues, TLR activation can lead to regional or systemic inflammation in the developing fetus. Future experiments will need to implement novel approaches for depleting specific fetal macrophage populations, as our antibody-based strategy was inefficient at cell depletion. Intrauterine fetal inflammation plays a major role in causing preterm birth and the long-term sequelae of extreme prematurity^53^. Inflammatory processes and tissue injury are associated with necrotizing enterocolitis^54^, bronchopulmonary dysplasia^55^, and retinopathy of prematurity^56^. If our data in mice translate to the development of human innate immunity, interventions targeting macrophage activation and inflammation processes will need to focus on specific pro-inflammatory macrophage populations.
